# Quantifying allele-specific CRISPR editing activity with CRISPECTOR2.0

**DOI:** 10.1093/nar/gkae651

**Published:** 2024-07-30

**Authors:** Guy Assa, Nechama Kalter, Michael Rosenberg, Avigail Beck, Oshry Markovich, Tanya Gontmakher, Ayal Hendel, Zohar Yakhini

**Affiliations:** Arazi School of Computer Science, Reichman University, Herzliya 4610101, Israel; The Institute for Advanced Materials and Nanotechnology, The Mina and Everard Goodman Faculty of Life Sciences, Bar-Ilan University, Ramat-Gan 5290002, Israel; The Institute for Advanced Materials and Nanotechnology, The Mina and Everard Goodman Faculty of Life Sciences, Bar-Ilan University, Ramat-Gan 5290002, Israel; Arazi School of Computer Science, Reichman University, Herzliya 4610101, Israel; Rahan Meristem (1998) Ltd. Kibbutz Rosh-Hanikra, Western Galilee 2282500, Israel; Rahan Meristem (1998) Ltd. Kibbutz Rosh-Hanikra, Western Galilee 2282500, Israel; The Institute for Advanced Materials and Nanotechnology, The Mina and Everard Goodman Faculty of Life Sciences, Bar-Ilan University, Ramat-Gan 5290002, Israel; Arazi School of Computer Science, Reichman University, Herzliya 4610101, Israel; The Henry & Marilyn Taub Faculty of Computer Science, Technion - Israel Institute of Technology, Haifa 3200003, Israel

## Abstract

Off-target effects present a significant impediment to the safe and efficient use of CRISPR-Cas genome editing. Since off-target activity is influenced by the genomic sequence, the presence of sequence variants leads to varying on- and off-target profiles among different alleles or individuals. However, a reliable tool that quantifies genome editing activity in an allelic context is not available. Here, we introduce CRISPECTOR2.0, an extended version of our previously published software tool CRISPECTOR, with an allele-specific editing activity quantification option. CRISPECTOR2.0 enables reference-free, allele-aware, precise quantification of on- and off-target activity, by using *de novo* sample-specific single nucleotide variant (SNV) detection and statistical-based allele-calling algorithms. We demonstrate CRISPECTOR2.0 efficacy in analyzing samples containing multiple alleles and quantifying allele-specific editing activity, using data from diverse cell types, including primary human cells, plants, and an original extensive human cell line database. We identified instances where an SNV induced changes in the protospacer adjacent motif sequence, resulting in allele-specific editing. Intriguingly, differential allelic editing was also observed in regions carrying distal SNVs, hinting at the involvement of additional epigenetic factors. Our findings highlight the importance of allele-specific editing measurement as a milestone in the adaptation of efficient, accurate, and safe personalized genome editing.

## Graphical Abstract

**Graphical Abstract:**
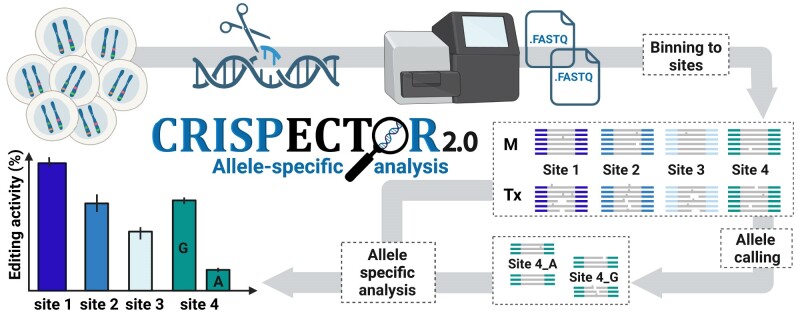

## Introduction

Clustered regularly interspaced short palindromic repeats (CRISPR)-Cas systems are rapidly gaining prominence in genome-editing research and related technology development, in humans and other organisms. However, this promising technology also gives rise to substantial ethical and technical challenges that necessitate attention (1–3). Among these challenges, off-target activity (OTA) represents a major concern, especially when CRISPR-Cas systems are being translated into the clinic (4). To edit a target locus, a guide RNA (gRNA) is delivered to the cell along with the CRISPR-Cas nuclease. OTA occurs when the gRNA/CRISPR-Cas complex binds to sites in the genome that differ from the desired target sequence, leading to unintended cleavage events. Ongoing efforts to mitigate OTA involve employing a combination of computational and molecular methods (5,6). Nevertheless, even with the implementation of these strategies, CRISPR-Cas can still significantly impact off-target sites, requiring a thorough in-depth characterization of off-target effects for every genomic target, in all applications where efficient and precise activity is desired.

Many assays and tools have been developed to nominate and characterize the experimented gRNAs, in terms of off-target sites and on-target editing frequencies (6–9). Bioinformatic prediction tools, such as COSMID (10) and Cas-OFFinder (11) are available for *in-silico* off-target site predictions. Additionally, several molecular-based assays enable the OTA assessment in a cellular context, such as GUIDE-seq (12) and DISCOVER-seq (13). Other cell-free methods utilized for off-target characterization include CHANGE-seq (14) and SITE-seq (15).

One method of quantifying the CRISPR-Cas9 activity at the off-target sites to assess their potency, in a cellular context, involves employing targeted multiplex PCR, using specifically designed primers (such as rhAmpSeq), to selectively amplify the putative OTA loci (16,17). In a previous study, we introduced CRISPECTOR, a powerful tool for analyzing multiplex PCR/next-generation sequencing (NGS) data and evaluating the nuclease activity at on- and off-target sites (18). CRISPECTOR utilizes a Bayesian classifier that detects editing activity even at low, yet significant, levels. By comparing CRISPR-Cas9-edited samples to unedited (mock) samples, CRISPECTOR calculates the probability of each event being attributed to the CRISPR-Cas9 editing and reports the possible error with associated confidence intervals (CIs). Moreover, CRISPECTOR incorporates a translocation feature, which identifies reads representing primer inconsistency and employs a hypergeometric statistical model to identify reads attesting to long insertions and deletions (INDELs) and/or translocations.

Genetic variation through single nucleotide variants (SNVs) is essential for species diversity and evolution. SNVs, which occur approximately every 1000 bp within the human genome, can alter protein function and individual phenotypes (19–21). The identification and characterization of these variations, known as allele variant calling, is vital when addressing therapeutic genome editing since even a single mutation within the anticipated target site or the protospacer adjacent motif (PAM) sequence can influence the gRNA–target interaction. The nucleotide alternation may augment or diminish the on-target editing efficiency and change the off-target profile of a specific patient or population (22–25), highlighting the need to thoroughly characterize the haplotypes of each potential patient and quantify the nuclease INDEL activity in an allelic context. Allele-specific editing can be also leveraged in the treatment of autosomal-dominant diseases, by targeting only the mutated allele (26–28). Allele variants are also abundant in plants, with crops frequently possessing a polyploid genome, including cotton, wheat, banana and many more (29). The presence of multiple alleles presents a challenge in genome editing within the field of agriculture, particularly when one aims to target all copies of a specific gene (30–32). In other cases, a partial knock-out (KO) of only specific alleles is needed, for instance when a complete KO would result in the plant's lethality (33). Overall, understanding and quantifying haplotype-dependent CRISPR activity, in both humans and plants, requires an assessment of the genome editing per allele.

Several tools and pipelines have been developed for analyzing CRISPR activity, among them are CRIS.py (34), BATCH-GE (35), CRISPR-GA (36), ampliCan (37) and CRISPR-A (38). CRISPResso2 (39) enables allele-specific quantification, however, it requires a separate reference for each allele, thus limited to primarily known SNVs.

In this study, we introduce CRISPECTOR2.0, an innovative tool designed to quantitatively assess CRISPR-Cas9 editing activity in an allele-specific manner. CRISPECTOR2.0 utilizes the control sample to identify nucleotides within the sequence that deviate from the reference, creating an individual-tailored reference sequence. In sites containing several SNVs, we employed an entropy-based statistical algorithm to call the haplo-alleles, taking into consideration both read frequency and read number. Finally, the NGS reads are allocated to the identified haplotypes and INDEL activity is determined for each allele separately. Overall, CRISPECTOR2.0 offers several advantages including (i) a statistical algorithm that effectively accounts for potential sequencing or amplification noise; (ii) *de novo*, per sample, SNV identification, eliminating the need for *a priori* information and (iii) precise allele-specific CRISPR-activity measurement. Hence, CRISPECTOR2.0 is the first tool that enables a reference-free, allele-aware measurement of on- and off-target activity.

## Materials and methods

### CRISPECTOR2.0 overview

CRISPECTOR2.0 is an easy-to-use software package with a command-line interface, which is an extension of CRISPECTOR software with an allele-specific option. Apart from the allele calling, all the other steps are based on the original tool and can be found in the CRISPECTOR publication (18). The input to the software is identical to CRISPECTOR and includes treatment (Tx) and unedited (mock) FASTQ input files, and a TXT format configuration file with the following fields, one for each site: site ID, gRNA sequence, amplicon sequence and primers. FASTQ files are first demultiplexed to different amplicons, and then a mock-based allele calling step follows. Tx and mock reads are then assigned to the alleles found. An INDEL quantification and translocation detection (which is not allele-specific) are performed as described before. Finally, the software outputs an HTML report containing detailed information regarding the INDEL frequency and pattern (both allele-specific and entire site), and translocation results.

### SNVs and allele calling

For each site, the mock reads are aligned to the amplicon sequence given by the user. Read length is determined by the majority of the reads and reads with a different length (‘diverse-length’ reads) are filtered and are not used for SNV identification. For each position, the entropy of the nucleotide frequency is calculated to determine whether it contains an SNV. Consider a position *t* on the amplicon. Let *p*(*t*) be the distribution of the observed symbols {A, C, G, T, –} at position *t*. We now compute the observed entropy of position t: $H( {p( t )} )$, wherein, for a distribution ${{p}_i} \ge 0$, $\mathop \sum \limits_{i = 1}^n {{p}_i} = 1$, the Shannon entropy is given by $H( p ) = - \mathop \sum \limits_{i = 1}^n {{p}_i} \cdot log{{p}_i}$.

If the entropy $H( {p( t )} ) \ge \tau$ (default value is τ = 0.72, representing a 20–80% ratio), then position *t* is called an SNV position. If several SNVs were detected along a single amplicon, all the SNV values of a single read are combined into one phased allele.

For haplo-allele determination, a potential allele distribution is calculated for all amplicon sites with at least one SNV. Let θ be all the possible phases (combinations) of SNVs in a given amplicon. We further denote $\theta = \{ {{{x}_1},{{x}_2}, \ldots ,{{x}_n}} \}$, and note that the number of possible phased SNVs satisfies ${{2}^k} \le n = len(\theta) \le {{5}^k}$, where k is the number of SNV positions detected for the amplicon.

Let ${{f}_i}$ be the frequency of the *i*th highest frequent nucleotide base / phase observed in the data. ${{f}_1} \ge {{f}_2} \ge {{f}_3} \ge \ldots \ge {{f}_n}$, such that $\mathop \sum \limits_{i = 1}^n {{f}_i} = 1$.

In most cases, we would not consider all the observed combinations as alleles, thus, the final selected alleles would satisfy:


\begin{eqnarray*}C\left( \nu \right) = \mathop \sum \limits_{i = 1}^\nu {{f}_i} \le 1\end{eqnarray*}

For these selected $\nu$ alleles, we can calculate the entropy as follows:


\begin{eqnarray*}H\left( \nu \right) = H\left( {\frac{{{{f}_1}}}{{C\left( \nu \right)}},\ \frac{{{{f}_2}}}{{C\left( \nu \right)}},\ \ldots ,\ \frac{{{{f}_v}}}{{C\left( \nu \right)}}} \right)\end{eqnarray*}

In order to determine the final number of alleles, we want to maximize both $C( \nu )$ and $H( \nu )$. However, in some cases, taking into consideration more frequencies of phased alleles results in smaller entropy. Thus, to control this tradeoff, we maximize the following expression, where ${{n}^*}$ is the final determined number of the alleles in the site:


\begin{eqnarray*}{{n}^*} = \mathop {argmax}\limits_{2 \le \nu \le n} \sum C{{\left( v \right)}^{\frac{1}{{10}}}}H{{\left( v \right)}^{\frac{9}{{10}}}}\end{eqnarray*}

Based on these results, ${{n}^*}$ new amplicons are created, all different from each other in at least one position and representing the haplo-alleles detected at the given site.

### Allocating reads to alleles

Following allele calling, all standard-length reads (reads identical in length to the reference amplicon) are assigned to the identified alleles using the discriminating SNVs identified in the SNV detection step. For ‘diverse-length’ reads the assignment is done by aligning each read to all possible ${{n}^*}$ new amplicons and the read is assigned to the amplicon that yields the best alignment score. The alignment step is described in the Supplementary Methods. For any of the candidates, if a matched alignment is found, then the read is allocated to the relevant allele. Else, it is classified as an ‘ambiguous read’. In some reads, the discernible SNV is either absent or contains a non-reference base (not associated with any allele). Initially, an attempt is made to rescue these ambiguous reads using a ‘multi-SNVs’ rescue strategy, relying on other non-masked linked-SNV. For each SNV, an alignment window is opened and computed against each of the alleles independently, as described before. If a higher alignment score is obtained for one allele, the read is assigned to that allele. In cases where only one SNV is present at the site or no linked SNV can be identified, the read is allocated randomly using the already observed proportions. Only sites with less than 50% random reads are subjected to further rounds of analysis, sites with higher percentages are discarded due to high ambiguity. See Supplementary Methods for a description of the random assignment process.

### Off-target amplicon sequencing in HEK293-Cas9 cells

#### Cell culture

Human Embryonic Kidney 293 cell line with stable expression of Cas9 protein (HEK293-Cas9 cells) was generated by Integrated DNA Technologies (IDT, Coralville, IA), as reported previously (40). HEK293-Cas9 cells were cultured in Dulbecco-modified Eagle Medium (DMEM) (Thermo Fisher Scientific, Waltham, MA, USA), supplemented with 10% fetal bovine serum (Thermo Scientific), 1% penicillin/streptomycin (Thermo Scientific), and 500 μg ml^−1^ G418 (Thermo Scientific).

#### GUIDE-seq

Off-target sites of 40 selected gRNAs (Supplementary Table S1) were nominated using GUIDE-seq (12), as described previously (16).

#### Electroporation

2-part gRNAs were produced via annealing of equal volumes of 200 mM 36 nt Alt-R 2-part XT crRNAs (IDT) and 200 mM 67 nt tracrRNA (IDT) in the final concentration of 100 mM annealed gRNA. After 5 min incubation at 95°C, the gRNAs were allowed to gradually cool down to room temperature. Electroporations were conducted as described previously (41). 0.3 × 10^6^ cells were electroporated with 4 mM annealed 2-part gRNA in a final volume of 25 ml inside a well of nucleocuvette strip (Lonza). Electroporated cells were plated on 6-well culture dishes in a 2 ml of complete medium. After 48 h, cells were harvested and subjected to genomic DNA extraction using QuickExtract DNA extraction solution (Lucigen, Middleton, WI, USA).

#### RNAse H-dependent PCR (rhAmpSeq)

rhAmpSeq assay (17) was conducted on the harvested DNA. The list of on- and off-target sites generated by GUIDE-seq software in a bed format using the hg38 reference genome was utilized as an input to rhAmpSeq Design Tool (https://eu.idtdna.com/pages/tools/rhampseq-design-tool) with default parameters. The resulting primer pools were used for a multiplex PCR on the 110 ng of genomic DNA according to the manufacturer's protocol. NGS library preparation was performed, as described previously (41). The sequencing data was generated on Illumina MiSeq, using MiSeq Reagent Micro Kit, v2 (300 cycles with 150 paired-end reads (Illumina, cat no. MS-103-1002).

### Off-target amplicon sequencing in banana cells

#### Banana (Musa acuminata) embryogenic cell suspension (ECS)

Banana ECS was used as a plant source for Agrobacterium-mediated transformation. ECS suspension was produced as described before (42), and maintained in ‘CS culture medium’ (1/2 MS, MS FeNaEDTA, 0.4 mg/l Thiamine, 0.5 mg/l Nicotinic acid, 0.5 mg/l Pyridoxine, 2.0 mg/l Glycine, 10 mg/l Ascorbic acid, 1.1 mg/l 2,4-d, 0.2 mg/l Zeatin, 3% sucrose). Five days after sub-culturing banana ECS were sieved through 400 μm nylon mesh, 0.1–0.2 ml of settled cell volume were aliquoted and resuspended in ‘CS culture medium’ to be used as starting material for further experiments.

#### Assembly of the transformation construct

The *M. acuminata* ‘DH-Pahang’ genome sequence was downloaded from the banana genome hub (https://banana-genome-hub.southgreen.fr/organism/1), and was used to design a target-specific gRNA (5′TGGAAGGCGGCGCCGCGGGA3′). For editing, a plant-specific binary vector (PmLBART) was modified to include TAcas9 (wheat (Triticum aestivum) codon-optimized version) downstream to the constitutive *Zea mays* ubiquitin promoter. Cestrum Yellow Leaf Curling Virus promoter was used for a gRNA expression. The modified vector was transformed into competent *Escherichia coli* (DH5-α) cells. Plasmid from *E. coli* clones was isolated and the sequences of the cas9 and gRNA were confirmed by Sanger sequencing (https://dna.macrogen.com/). The positive vector was transformed into the EHA105 strain of Agrobacterium tumefaciens by electroporation. Bacterial culture and banana transformation are described in Supplementary Methods.

#### Amplicon library prep and sequencing

Genomic DNA was extracted from the ECS 10 weeks after transformation, with genomic DNA mini kit (plant) (GENEAID Biotech Ltd. Taiwan). Total DNA was used to amplify the desired amplicons across the different targets with unique primer pairs (Supplementary Table S2). PCR was performed using 20–40 ng genomic DNA template, 0. 5 μM primers, and 2× Platinum SuperFI II PCR Master mix (Invitrogen), in a total reaction volume of 20 μl. Cycling conditions for PCR were as follows: initial denaturation at 98°C for 1 min, followed by 35 cycles of denaturation at 98°C for 5 s, annealing at 60°C for 30 s, and extension at 72°C for 10 s, final extension at 72°C for 2 min. Library preparation and sequencing were conducted by the Technion Genomics Center (Haifa, Israel). Libraries were constructed simultaneously according to Illumina 16S Metagenomic Sequencing Library Preparation. The sequencing data was generated on Illumina MiSeq, using MiSeq Reagent Micro Kit, v2 (300 cycles with 150 paired-end reads (Illumina, cat. no. MS-103-1002).

### CRISPECTOR2.0 running configurations

HEK293-Cas9 paired-end FASTQ files were analyzed with CRISPECTOR2.0 using bioconda (https://anaconda.org/bioconda/) environment with default parameters. *CD33_846* and *NR3C1_KO1* off-target panel can be found in Supplementary Table S3 and Supplementary Table S4, respectively.

Banana paired-end FASTQ files were analyzed with CRISPECTOR2.0 with the following running parameters: –fastp_options_string ‘–overlap_len_require 10 –overlap_diff_limit 6 –overlap_diff_percent_limit 30’.

Primary cells data was downloaded from NCBI and analyzed in CRISPECTOR2.0 with default parameters.

## Results

### CRISPECTOR2.0 is a tool for allele-specific CRISPR-Cas9-activity quantification

**Figure 1.**
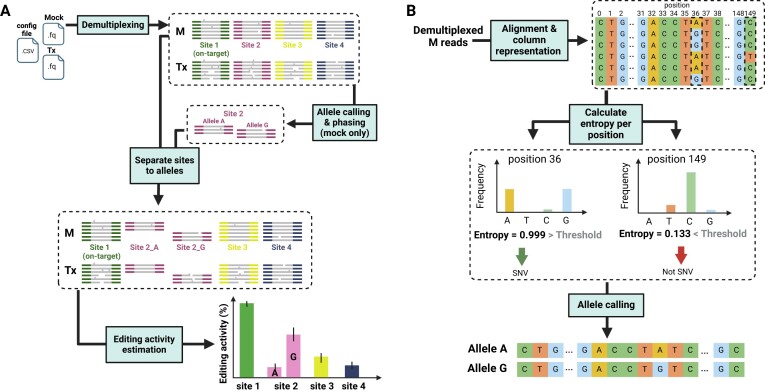
CRISPECTOR2.0 is a tool for allele-specific quantification of on- and off- target CRISPR activity in multiplex PCR NGS data. (**A**) Schematic description of the CRISPECTOR2.0 pipeline. Tx and mock (M) reads are first demultiplexed to the different panel sites. Next, CRISPECTOR2.0 identifies SNVs and conducts site-specific phasing using the mock sample as reference. M and Tx reads are then assigned to their respective alleles, and INDEL quantification is done per allele. (**B**) Illustration of CRISPECTOR2.0 allele calling algorithm. To find SNVs, CRISPECTOR2.0 first aggregates the M reads and calculates the nucleotide content in each position. Positions with entropy above a specified threshold are subsequently called as SNV positions. Phasing is then performed utilizing an approach that considers both the entropy and read number in each potential haplotype. For further details, see Materials and methods.*Created with BioRender.com*.

CRISPECTOR2.0 is an extended version of CRISPECTOR, offering the same precise genome-editing quantification algorithm while also enabling the quantification of on- and off-target activity in an allele-specific manner. The software utilizes both unedited (mock) and treatment (Tx) FASTQ files, along with a configuration file containing experiment parameters and details, as input. CRISPECTOR2.0 first assigns the Tx and mock reads to the target genomic sites. Next, for each site, CRISPECTOR2.0 processes the mock reads and looks for SNVs. If found, CRISPECTOR2.0 performs allele calling and then splits the Tx and mock reads into the identified alleles. Finally, the workflow produces a summary of INDELs for each characterized allele distinctively. To facilitate user interpretation and further analysis of the results, CRISPECTOR2.0 generates a comprehensive HTML-based report (Figure 1A).

A key feature of CRISPECTOR2.0 is the SNV detection and allele-calling, performed without the need for a known reference, and instead using the wild-type mock sample to identify SNVs *de novo*, as demonstrated in Figure 1B. CRISPECTOR2.0 utilizes an entropy-based model (see Materials and methods) to distinguish between true putative SNVs and artifacts, such as noise generated during NGS library preparation or sequencing, thereby enhancing the accuracy of the results. Next, an additional entropy-based model determines the haplo-alleles in the sample, at each interrogated site, taking into account both the population size (read number) and the inferred allele frequency. Furthermore, the model assumes SNV independence, enabling the identification of a potentially unlimited number of alleles. The allele number flexibility is advantageous in complex genetic scenarios, such as polyploid organisms, or cells with aberrant chromosome copy number characteristics.

### CRISPECTOR 2.0 identifies SNVs *de novo*

**Figure 2:**
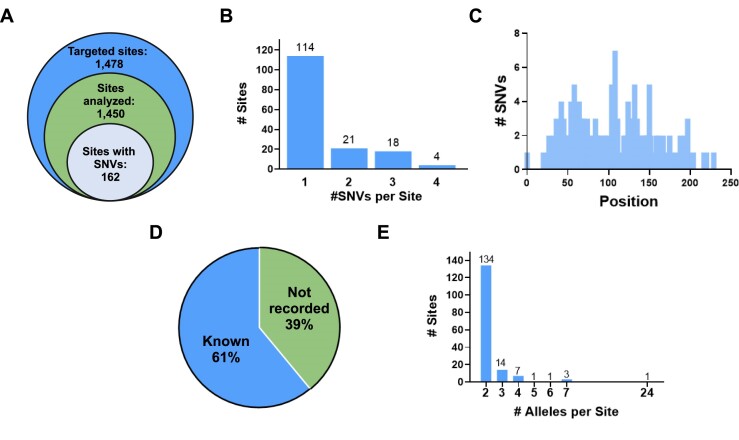
*De novo* SNV calling and phasing. (**A**) A summary of the allele analysis conducted in this study is as follows. We commenced the analysis with 1478 on- and off-target sites originating from 40 distinct gRNAs (outer circle). Out of these, 1450 sites were analyzed (middle circle), and among them, 162 sites contained SNVs (inner circle). (**B**) We have found a total of 244 SNVs in our dataset, with some sites containing multiple SNVs (up to 4 SNV positions per site). (**C**) The distribution of the identified SNV positions spans the entire amplicon, with amplicon sizes ranging from 150 to 250 bp. (**D**) 39% of the detected SNVs had not been previously reported in published datasets (blue). (**E**) Upon allele calling, the tested sites contained a diverse range of allele numbers, with the majority being in a diploid state. *Created with BioRender.com*.

To evaluate the efficacy of CRISPECTOR2.0 allele calling algorithm, we conducted extensive tests on a vast dataset consisting of amplicon sequencing of 1478 on- and off-target sites of 40 gRNAs, performed on HEK293-Cas9 cell line samples (see Supplementary Table S1 for a detailed list of all gRNAs). The stable Cas9 overexpression enhances measurement sensitivity and enables improved OTA detection, effectively capturing rare events that might be missed due to the NGS limitations. Using CRISPECTOR2.0, we successfully identified 244 SNVs in 162 different sites, with a maximum of four SNVs observed at a single amplicon. Overall, 10% of the sites contained at least one SNV (Figure 2A, B). The SNVs were uniformly distributed across the entire amplicons, both upstream and downstream from the anticipated CRISPR-Cas9 cleavage site, indicating that the algorithm is not position-biased (Figure 2C*)*. We then validated the detected SNVs by comparing them to dbSNP (43) and to the 1000 Genome Project (44) database. We found that only 61% of our SNVs were previously reported by any of these datasets (Figure 2D, Supplementary Table S5). This means that we detected the presence of 39% formerly unreported SNVs. These sites are potentially sample-specific, highlighting the importance of *de novo* SNV detection, which does not rely on database information.

Following the SNV detection phase, CRISPECTOR2.0 proceeds with allele calling, calculating the frequency of the different alleles to determine the haplotype structure of the sample, for each amplicon. Allele calling becomes particularly challenging in cases where multiple SNVs reside on the amplicons, and additional complexity arises from the presence of sequencing and amplification noise. Nevertheless, CRISPECTOR2.0 had discerned up to seven distinct haplo-alleles per amplicon site (Figure 2E), thereby highlighting the usefulness of its allele calling feature. Following allele calling, each read can be accurately assigned to its corresponding haplotype, thereby facilitating the subsequent allele-specific quantification step.

During the process of assigning reads to alleles, there are instances in which the SNV distinguishing base is masked, due to noise aggregated in the library preparation or sequencing processes, or nuclease-induced INDELs in the treated reads (herein referred as ‘ambiguous reads’). In alleles harboring two or more SNVs, the presence of another ‘anchor’ SNV in the amplicon can rescue the ambiguous read, allowing for confident assignment. In other cases, a deterministic assignment is unattainable. Omitting such reads will result in low coverage and skew the results, particularly when an SNV lies near the nuclease target site and hence is deleted in many of the treated reads. To address the ambiguous reads, CRISPECTOR2.0 first analyzes each sample without any of the ambiguous reads and quantifies the editing activity. Next, CRISPECTOR2.0 iteratively performs ‘random assignment’, allocating reads to alleles in proportions aligned with the distribution of alleles in the mock, and with the confidently assigned treatment reads. Finally, CRISPECTOR2.0 reports the medoid editing activity results, along with upper and lower bounds (Supplementary Figure S1). This methodology not only ensures the inclusion of all reads but also allows a confident yet conservative editing activity estimation.

### Differential allelic editing at on- and off-target sites

**Figure 3.**
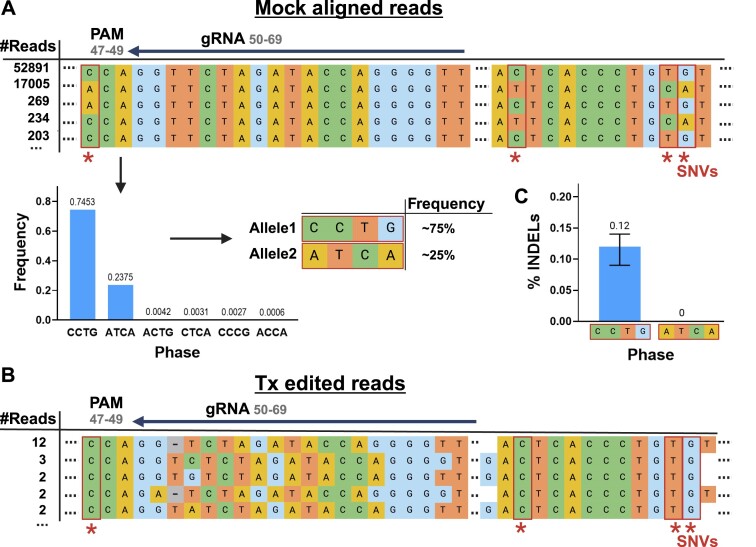
SNV in the PAM sequence resulted in off-target allele-specific CRISPR activity. Illustration of CRISPECTOR2.0 pipeline results for the off-target site *CD33_846_33* of a gRNA targeting the *CD33* gene (*CD33_846*) (57). (**A**) In the SNV detection step, four distinct SNVs were identified, including one located within the PAM sequence at position 47 within the amplicon. Subsequently, two alleles were called: a PAM-preserved allele (*‘CCTG’*) and a PAM-disrupted allele (*‘ATCA’*), with read frequencies of 75% and 25%, respectively. (**B**) Edited Tx reads, from the PAM-preserved *CCTG* allele, as identified by CRISPECTOR2.0. (**C**) CRISPECTOR2.0 had found INDELs only in the *CCTG* allele (editing activity = 0.12%, 0.95CI = [0.09%, 0.14%]), while in the PAM-disrupted *ATCA* allele, we observed no editing activity (editing activity = 0%). *Created with BioRender.com*.

Allele variance can affect the editing activity, especially when there are alterations in the gRNA target sequence or the adjacent PAM motif. Using CRISPECTOR2.0 allele-sensitive version, we identified sites with variations in editing activity among different alleles. Notably, we detected an off-target site of a guide targeting the *CD33* gene containing an SNV in the PAM sequence, resulting in one allele with a disrupted PAM sequence, and another with a preserved PAM sequence. Consequently, only the PAM-preserved allele had detectable CRISPR-Cas9 editing, consistently over two replicates (Figure 3).

**Figure 4.**
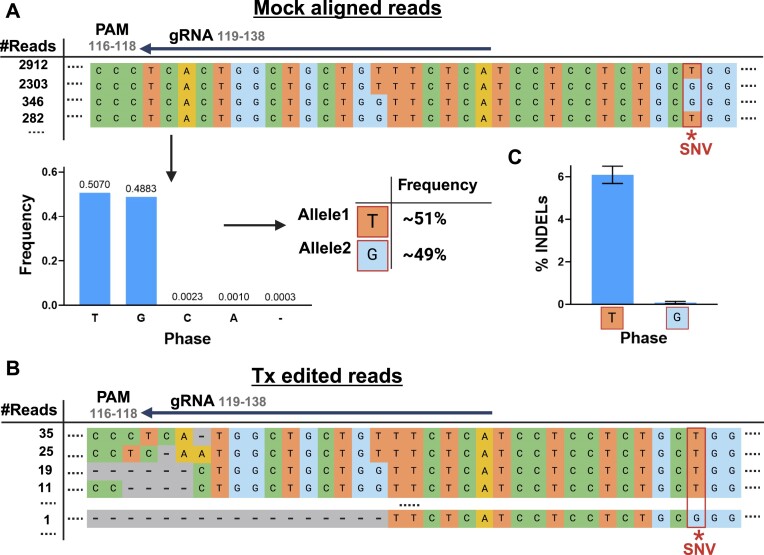
Distal SNVs in off-target allele-specific CRISPR-Cas9 activity. Illustration of CRISPECTOR2.0 pipeline results for the off-target *NR3C1_KO_6* of a gRNA targeting the *NR3C1* gene (*NR3C1_KO*). (**A**) CRISPECTOR2.0 identified a *T/G* SNV 12 bp upstream of the protospacer sequence. (**B**) Despite the distance from the nuclease recognition site, we observed differential editing as most of the edited Tx reads identified by CRISPECTOR2.0 originated from the *‘T’* allele. (**C**) INDEL frequency in each of the alleles as quantified by CRISPECTOR (‘*T*’ allele: editing activity = 6.09%, 0.95CI = [5.68%, 6.49%]; ‘*G*’ allele: editing activity = 0.08%, 0.95CI = [0.03%, 0.14%]). *Created with BioRender.com*.

In another off-target site of a gRNA targeting the *NR3C1* locus (45), we observed a 6% difference in INDEL frequency between the two alleles, although the SNV position is almost 30 bp downstream from the cut-site (Figure 4). These results indicate the importance of considering distal SNVs when measuring genome editing activity outcomes, as they may affect or be correlated with editing activity levels at the cleavage site. In both cases, the editing activity reported by the previous allele-indifferent CRISPECTOR version masked the true editing-activity values, as the editing activity of the more extensively edited allele consistently surpassed the allele-indifferent value. These findings demonstrate that when quantifying the editing activity of a genomic locus with more than one allele, the allele-indifferent approach not only ignores the variability between alleles but may also result in a lower reported editing activity than should be considered.

### Allele-specific differential editing activity in human primary cells

**Figure 5.**
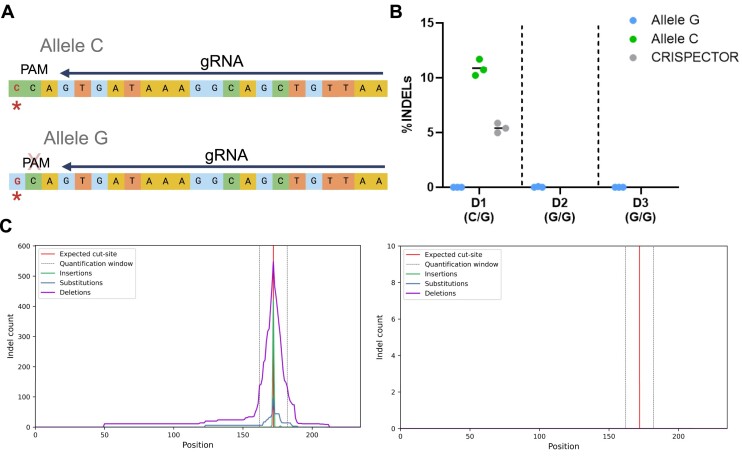
Off-target allele-specific editing in human primary cells. CRISPECTOR2.0 results on an off-target site for a gRNA (#1617) targeting the *BCL11A* enhancer (**A**) Illustration of the chr2:210530659–210530681 genomic locus harboring a *C/G* SNV (marked by a red asterisk). The *‘C’* allele generates a 5′-NGG-3′ PAM sequence aligned with the gRNA. (**B**) Editing activity results for three donors: a heterozygous donor (D1) and two homozygous donors (D2-D3), both carrying the no-PAM SNV. In D1, we observed selective editing in the PAM-containing *C* allele (mean: *C*: 10.87%, *G*: 0%, *n* = 3). In the other two homozygous donors, no editing was observed (D2: mean: 0%, *n* = 3, D3: mean: 0%, *n* = 3). Tabulated data can be found in Supplementary Table S6. (**C**) Edited events distribution for the heterozygous donor, in the *C* allele (*left*) and in the *G* allele (*right*). *Created with BioRender.com*.

The previous findings were obtained from the HEK293-Cas9 cell line, which, while enabling a more sensitive OTA detection, does not accurately represent the normal human genome due to numerous chromosomal aberrations (46). To evaluate the performance of our tool in primary human cells, we analyzed CD34+ hematopoietic stem and progenitor cells (HSPCs) data from a study conducted by Cancellieri *et al.* (47). In this study, a C/G SNV (rs114518452) resulted in a 5′-NGG-3′ PAM sequence motif, which serves as an off-target site of a gRNA targeting the *BCL11A* enhancer (48). We tested CRISPECTOR2.0 on amplicon sequencing data of the three individuals reported in the original article, with one found to have a heterozygous C/G genotype, harboring a functional PAM sequence in one allele, and two homozygous G/G donors, without any PAM sequence at the tested locus. CRISPECTOR2.0 identified the two alleles, and, in line with the original publication, revealed selective editing activity exclusively for the *C* allele. The G allele showed no editing activity, across all three human samples (Figure 5, Supplementary Figure S5).

### Quantifying allelic editing in polyploid agriculture crops

**Figure 6.**
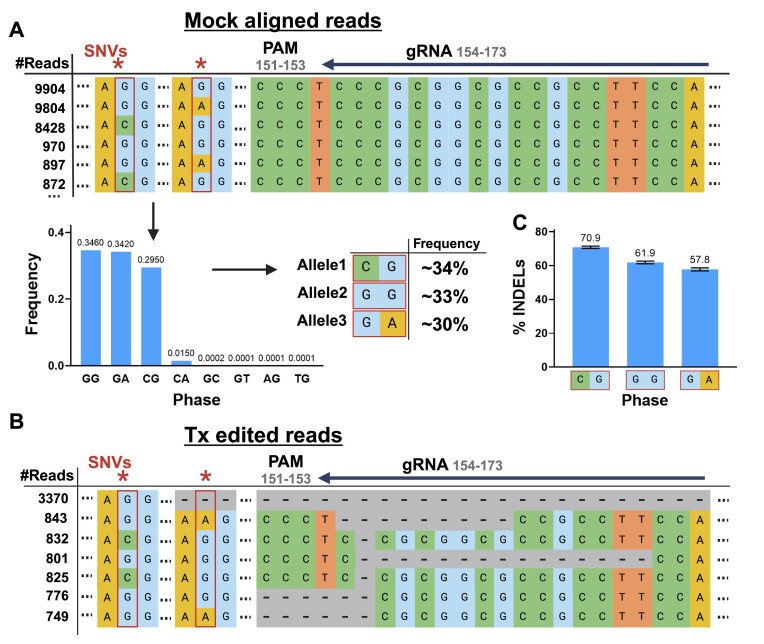
Validation of multi-allelic editing in *Musa acuminata*. The CRISPECTOR2.0 pipeline results are presented for a gRNA targeting the *DHS* gene. (**A**) During the SNV detection step, two distinct SNVs were identified downstream of the PAM sequence in the on-target site. Three alleles were called, with respective allele frequencies of 31%, 34% and 35%. SNV bases are marked by a red asterisk. (**B**) Edited reads were detected in all three alleles, indicating successful editing. (**C**) All three alleles exhibited comparable editing activity, with mean editing percentages of 70.9%, 61.9% and 57.8% and corresponding CIs as indicated. *Created with BioRender.com*.

In the field of agricultural genome editing, where many organisms possess polyploid genomes, it becomes imperative to either edit all alleles or selectively focus on specific alleles (for example to mitigate potential lethality). In both scenarios, CRISPECTOR2.0 provides a means to confirm the accuracy of on-target editing across the different alleles and to verify that all desired alleles were, in fact, edited. To assess the tool's performance in analyzing plant genomes, we conducted experiments in *Musa acuminata*, a banana species characterized by a triploid genome. Specifically, we evaluated the on-target editing efficiency of a gRNA designed to knock out all three copies of the *Deoxyhypusine synthase* (*DHS*) gene. In this site amplicon, CRISPECTOR2.0 detected two SNVs located downstream of the PAM sequence, revealing the presence of three different alleles with various combinations of these two SNVs, in line with the three chromosome copies of the banana (Figure 6A, B). INDEL rate was high in all three alleles (71.03%, 61.47% and 57.89% INDELs), providing evidence of effective locus editing but also pointing to potential differences (Figure 6C).

## Discussion

This study demonstrates the effectiveness of CRISPECTOR2.0 as a tool for precise quantification of allele-specific editing activity in CRISPR-Cas9 experiments. We first showcased the performance of the SNV identification feature by analyzing data from the HEK293-Cas9 cell line, successfully detecting SNVs in more than 200 sites within the interrogated amplicons in a reference-free manner. We further demonstrated CRISPECTOR2.0's allele-specific editing activity quantification capabilities in sequencing data originating from cell-line, primary cells, and polyploid organisms. Finally, we described several cases in which editing activity was restricted to one allele. This phenomenon is typically observed when the protospacer or PAM sequences carry an SNV, thus affecting the nuclease binding. Nevertheless, we showed that differential editing activity also occurs when the sequence variation resides outside of the 23 bp target region defined by PAM + protospacer.

CRISPECTOR2.0 offers several key features that enhance its precision. First and foremost, the SNV identification and editing activity quantification in CRISPECTOR2.0 are based on the control sample. While users provide an initial reference sequence for read assignment to specific sites, the sequences from the control sample function as a *de facto* reference sequence for SNV calling, and serve as a negative control when quantifying activity, thus reducing false positive counts attributable to sample-specific background or sequencing noise. In addition, CRISPECTOR2.0′s allele-specific analysis identifies instances where variations in editing activity occur between alleles, or where a single allele undergoes exclusive editing. This analysis holds particular significance when targeting dominant mutations, for example in severe congenital neutropenia and Huntington's disease (49). Genome-editing strategies for these mutations require a complete and specific knock-out of the mutated allele, and CRISPECTOR2.0 can serve as a tool to validate these results. Detecting and quantifying allele-specific editing activity is also crucial in polyploid organisms, such as those in the agriculture industry. In scenarios where multiple alleles or genes must be simultaneously targeted, there is a need for precise validation of intended knock-in or knock-out results. The ability to handle any number of alleles, the *de novo* variant calling feature, and the sensitive INDEL quantification offered by CRISPECTOR2.0 position it also as a powerful solution for quantifying genome editing products in agriculture.

Although CRISPResso2 (39) enables SNV quantification based on prior knowledge, our results show that relying on existing databases is insufficient, as the presence of non-reported SNVs introduces biases in editing activity. Since we validated CRISPECTOR2.0 using the HEK293-Cas9 cell line, it is plausible that specific ‘formerly-unreported’ SNVs are unique to this particular cell line, and the proportion of novel SNVs in primary cells is expected to be lower. However, even the existence of a single SNV might have deleterious effects, and in clinical applications, the SNV profile of each patient must be carefully addressed. Moreover, in organisms other than humans, particularly in agricultural crops, hybrid species or limited data availability restrict the use of pre-existing references. Therefore, the variant calling step of CRISPECTOR2.0 is important in empowering precision genome editing across diverse cell types.

A variety of tools and algorithms have been developed for the purpose of haplotype separation (50). In our study, we sought to employ the mock sample for haplotype calling, yielding several notable advantages: (i) precision at the individual level; (ii) simplicity and (iii) independence from the apriori data. As our analysis focuses on amplicon sequencing, it is possible that some of the called SNVs are, in fact, aggregates of PCR artifacts occurring during the early stages of amplification. Given that false positive SNVs do not introduce bias in editing activity, they have no significant impact on the analysis. Strategies such as UMI-PCR (Unique Molecular Identifier PCR) can be employed to further enhance the sensitivity and accuracy of the allele-calling step (51,52).

In the context of genome-editing clinical trials, edited patient cells are expanded ex-vivo to obtain a sufficient number of input cells. In the experiments and measurements of this manuscript, the off-target analysis was conducted on untreated and treated cells undergoing expansion side-by-side for the same period of time. In clinical trials the reference cells might be sourced from the initial patient sample prior to the cell expansion. In such cases, potential negative or positive selection of a specific allele in the expanded treated sample will bias the allele frequencies. Since CRISPECTOR2.0 uses a comparative Bayesian test to discern real editing events from artifacts, an imbalanced read number for a given allele in the treated and mock samples can lead to effect size issues in the analysis. While allele phasing, which is done solely based on the mock sample, would remain unaffected, editing activity rates may be slightly deflected, when small read numbers are involved. In such cases, CRISPECTOR2.0 will inform the user that data is imbalanced, and caution should be exercised in interpreting the results (see Supplementary Methods). Having analyzed 1478 sites in this study, allele frequency imbalance was only observed in one unedited off-target site, as indicated in Supplementary Table S7. Despite the rarity of these observations, the user may select to repeat the measurement when an allele frequency imbalance occurs.

The presence of a robust editing-activity difference between alleles often arises when an SNV disrupts or creates a functional PAM motif, resulting in editing limited to the allele harboring the functional PAM site. Our data provided further evidence of this phenomenon, demonstrating that heterozygous genotypes exhibit differentially edited alleles. CRISPRme (47) can predict such cases by pre-designing gRNAs in an allele-aware fashion. However, this tool does not offer solutions for SNVs that are unknown or located outside the protospacer or PAM region. Given the current limitations of predictive models, we highly advise conducting individual genome testing as the preferred approach. This personalized strategy allows for a comprehensive, precise, and detailed evaluation of the unique genomic context, facilitating a more accurate assessment of potential off-target effects.

Interestingly, one SNV in our study that was associated with differentially edited alleles was located 30 bp from the cut site (as depicted in Figure 4). The distal-SNV site showed pronounced disparities in editing activity, to the extent that one allele was exclusively edited. These findings imply the potential influence of additional genomic factors, such as chromatin structure, DNA methylation, or other epigenetic properties, on the affinity and efficacy of nuclease cutting. It is well documented that the epigenetic factors correlate to the off-target activity, so that genomic targets in open-chromatin regions, in proximity to highly transcribed loci or active promoters, are more prevalent in off-target editing (14,53,54). Since CRISPECTOR2.0 analysis is focused on SNVs within the short range of the NGS amplicon, we cannot determine based on our findings whether the identified SNV itself caused the differential editing or other *cis* or trans elements associated with the discerned allele. Further studies should be conducted to address the correlation between remote SNVs and linked epigenetic factors, integrating long-read sequencing techniques to capture the entire genomic landscape of the haplotypes, and leveraging epigenetic sequencing methods, such as ChIP-seq (55) or ATAC-seq (56).

In summary, through the integration of allele-specific quantification, CRISPECTOR2.0 emerges as a potentially useful and efficient tool for the precise and detailed analysis of NGS amplicon CRISPR experiments across diverse organisms. This cutting-edge tool not only enhances the precision and resolution of CRISPR-Cas9 off-target measurements but also represents an important step toward achieving personalized, accurate, and safer genome editing.

## Code availability

CRISPECTOR2.0 command line tool is available as a Python pip package at https://pypi.org/project/crispector2, or as a Bioconda package at https://anaconda.org/bioconda/crispector2. CRISPECTOR2.0 source code, installation and user manual are available online at https://github.com/YakhiniGroup/crispector2 and https://doi.org/10.6084/m9.figshare.26152804.

## Supplementary Material

gkae651_Supplemental_Files

## Data Availability

The sequencing data obtained from HEK293-Cas9 and banana cells, containing sites with differential editing, have been deposited in the National Center for Biotechnology Information (NCBI) under accession number: PRJNA998321. The sequencing data of the *BCL11A* gRNA off-target site (containing the rs114518452 SNV) from Cancellieri *et al.* (47) was obtained from the NCBI repository under accession number: PRJNA733110. Specific SRA files used for CRISPECTOR2.0 analysis are detailed in Supplementary Figure S6. dbSNP and 1000G data were obtained from https://www.ncbi.nlm.nih.gov/snp/. All other data generated in this project is available from the corresponding authors upon request.
