# Supplementary material for: Quantifying allele-specific CRISPR editing activity with CRISPECTOR2.0
Source: Nucleic Acids Res. 2024 Jul 30;52(16):e78. doi: 10.1093/nar/gkae651 (PMC11381363; doi:10.1093/nar/gkae651)
Supplement: gkae651_Supplemental_Files [file gkae651_supplemental_files.zip › Supplementary Data 240624 2nd revision.pdf]

SUPPLEMENTARY FIGURES

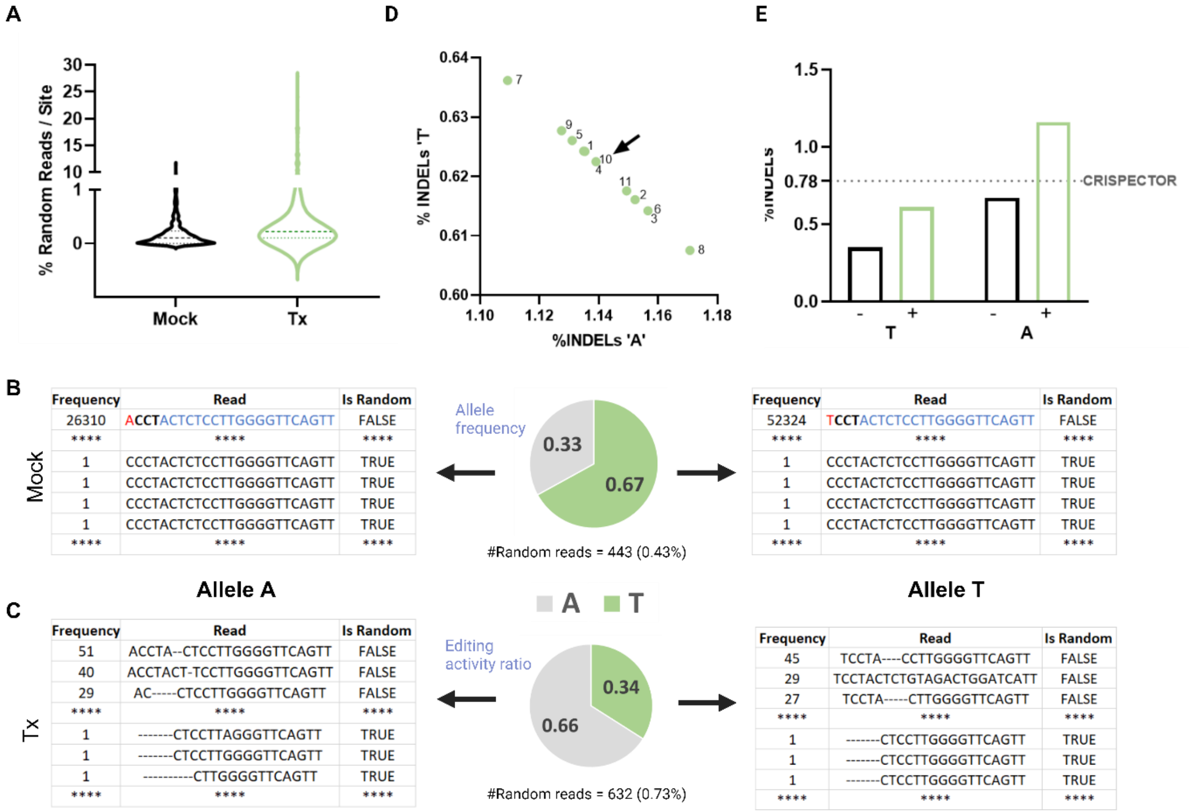

Supplementary Figure 1: Allocation of ambiguous reads via random assignment. (A) Number of random reads across all the HEK293-Cas9 experiments tested (n=80), in the Mock and Tx samples. Dashed lined represent 25th percentile (bottom line, Mock=0%, Tx=0.1%), median (middle line, Mock=0.1%, Tx=0.22%), and 75th percentile (top line, Mock=0.23%, Tx=1.65%). **(B-E)** Demonstration of a random assignment cycle in the *RAG1_12* off-target site, containing an SNV downstream the PAM sequence. **(B)** One cycle of random read allocation process in *RAG1_12* Mock sample. A total of 443 reads were allocated via random assignment according to the allele frequency, shown in the pie graph (Allele 'A': 0.33, Allele 'T': 0.67). Left and right tables show reads assigned to the 'A' and 'T' alleles, respectively. For each allele, the reference sequence is shown in the upper line. Red base represents the SNV base, bold bases represent the PAM sequence, and blue bases represent the protospacer sequence. Ambiguous reads, in which the SNV base is masked, can be identified via the "TRUE" value in the 'Is Random' field. **(C)** One cycle of random read allocation process in *RAG1_12* Tx sample. 632 reads were randomly allocated using an 'editing activity coin', which represents the ratio of editing activity between alleles per site before the random assignment ('A': 0.66, 'T': 0.33). Demonstration of some of the assigned reads to each of the alleles can be shown in the left and right tables, respectively. **(D)** Editing activity calculated for each allele in each of the 11 random assignment rounds. The medoid value, reported as the final editing activity, is marked with a black arrow ('A'=0.61%, 'T'=1.16%). **(E)** Reported editing activity values for

RAG1_12, before or after random assignment (black and green bars, respectively) (Before: ‘A’=0.34%, ‘T’=0.66%; After: ‘A’=0.61%, ‘T’=1.16%). CRISPECTOR allele-unaware editing activity is marked with a dashed line (%INDELs=0.78).

SUPPLEMENTARY METHODS

Alignment of Reads to Reference Alleles

A 20bp window (10bps on each site) is created around the SNV position. Next, the sequence window is aligned against ‘diverse-length’ reads to try and rescue the SNV base: for SNVs that are <10 bps from the cut-site a ‘local strict alignment’ is used (match_score: 5, mismatch_score: 1,open_gap_score: -100, extend_gap_score: -100, target_end_gap_score: 0.0, query_end_gap_score: 0.0), otherwise a ’local loose alignment’ is used (match_score: 5, mismatch_score: -4, open_gap_score: -10, extend_gap_score: 0, target_end_gap_score: 0.0, query_end_gap_score: 0.0).

Random Reads Allocation

Before the random assignment, an initial round is performed without the ambiguous reads to estimate the editing activity. Ambiguous reads are then assigned based on the following principles: Ambiguous mock reads are randomly assigned to their alleles based on a "coin" flip with the probability of the allele's frequency. Similarly, ambiguous treatment reads are assigned in a comparable manner, but with a "coin" bearing the probability of the allelic editing activity ratios. The random assignment and editing activity calculation are repeated an additional 10 times, for a total of 11 random cycles. At the end of these cycles, the medoid result of the editing activity is reported, along with the highest and lowest CIs computed.

Imbalanced Allele Frequency

To identify sites with inconsistent mock/tx frequencies across alleles, we employed Jensen-Shannon Divergence (JSD). We further calculated the absolute difference between the mock and tx ratios for each allele (Nominal Ratios Gap (NRG)). Sites were classified as having imbalanced read numbers based on one of two criteria: JSD>=0.2, or a combined threshold of JSD>=0.1 and NRG>=2.

EHA105 Bacterial Culture

Agrobacterium tumefaciens strain EHA105 containing the genome editing vector was incubated on liquid 2YT medium for 48 hrs at 27-29°C. Cells were pelleted by centrifugation at 4,000 rpm for 10 min, resuspended in ‘bacterial resuspension medium’ (1/10 MS, 9 mg/L Thiamine, 0.4 g/L Cysteine, 3.6% glucose, 6.85% sucrose, 100 µM Acetosyregone) and adjected to OD600 0.3-0.6.

Banana Transformation

Banana ECS was incubated at 45°C for 5 min and was added with 0.8-1.0 ml of pre-induced bacterial culture. The combined induced bacteria and the cell suspension were centrifuged at 1,000 rpm for 5 min at R.T and co-incubated for 25 min. Excess bacteria was then removed from ECS, which was followed by resuspension of ECS in a fresh 'CS culture medium'. ESC was then transferred to a sterile glass microfiber filter and was placed in Petri dishes (55 mm) with 'co-cultivation medium' ('CS culture medium' solidified with 0.25% gelrite (Duchefa) and supplemented with 100 µM Acetosyrengone) for 3 days. Agro-infected ECS were allowed to proliferate in 'CS culture medium' solidified with 0.25% gelrite (Duchefa) and supplemented with 300 mg/L Cefotaxime. Putatively transformed cells were transferred to 'CS culture medium' solidified with 0.25% gelrite (Duchefa) supplemented with 300 mg/L Cefotaxime and 10 mg/L Hygromycin, and sub-cultured every 4 weeks.

SUPPLEMENTARY TABLES

Supp. Table 1. List of all gRNAs used in the HEK293-Cas9 experiments.

Supp. Table 2. Primer-pair used for amplification of the *DHS* gene in *M. acuminata*.

Supp. Table 3. rhAmpSeq panel used for amplification of the *CD33_846* gRNA's off-target sites.

Supp. Table 4. rhAmpSeq panel used for amplification of the *NR3C1_KO1* gRNA's off-target sites.

Supp. Table 5. List of all SNVs identified in the HEK293-Cas9 experiments.

Supp. Table 6. Tabulated data of figure 5B, showing INDEL rate of primary-cell donors, at an off-target site of the *BCL11A* gRNA.

Supp. Table 7. Sites with imbalanced allele ratio.
